# Distal neuropathic pain in HIV is associated with functional connectivity patterns in default mode and salience networks

**DOI:** 10.3389/fpain.2022.1004060

**Published:** 2022-10-12

**Authors:** Chelsea C. Hays Weeks, Alan N. Simmons, Irina A. Strigo, Sara Timtim, Ronald J. Ellis, John R. Keltner

**Affiliations:** ^1^ CESAMH, VA San Diego Healthcare System, San Diego, United States; ^2^ Department of Psychiatry, UC San Diego, La Jolla, CA, United States; ^3^ Department of Psychiatry, UC San Francisco, CA, United States; ^4^ Department of Neurosciences, UC San Diego, La Jolla, CA, United States

**Keywords:** HIV, neuropathic pain, fMRI, functional connectivity, default mode network

## Abstract

HIV-associated distal neuropathic pain (DNP) is one of the most prevalent, disabling, and treatment-resistant complications of HIV, but its biological underpinnings are incompletely understood. While data specific to mechanisms underlying HIV DNP are scarce, functional neuroimaging of chronic pain more broadly implicates the role of altered resting-state functional connectivity within and between salience network (SN) and default mode network (DMN) regions. However, it remains unclear the extent to which HIV DNP is associated with similar alterations in connectivity. The current study aimed to bridge this gap in the literature through examination of resting-state functional connectivity patterns within SN and DMN regions among people with HIV (PWH) with and without DNP. Resting state functional magnetic resonance imaging (rs-fMRI) scans were completed among 62 PWH with HIV-associated peripheral neuropathy, of whom 27 reported current DNP and 35 did not. Using subgrouping group iterative multiple estimation, we compared connectivity patterns in those with current DNP to those without. We observed weaker connectivity between the medial prefrontal cortex (MPFC) and posterior cingulate cortex (PCC) and stronger connectivity between the anterior cingulate cortex (ACC) and thalamus among those reporting DNP. Overall, these findings implicate altered within DMN (i.e., MPFC-PCC) and within SN (i.e., ACC-thalamus) connectivity as potential manifestations of adaptation to pain from neuropathy and/or mechanisms underlying the development/maintenance of DNP. Findings are discussed in the context of differential brain response to pain (i.e., mind wandering, pain aversion, pain facilitation/inhibition) and therapeutic implications.

## Introduction

Since the introduction of combination antiretroviral treatment (CART) to treat HIV more than 20-years ago, HIV-associated distal neuropathic pain (DNP) has emerged as one of the most prevalent, disabling, and treatment-resistant complications of this condition (1). Affecting more than 20% of the HIV + population and typically refractory to CART and chronic pain therapies, HIV DNP is associated with unemployment, functional decline, and overall reduction in quality of life (1, 2). Improved mechanistic understanding of HIV DNP could lead to development of treatments aimed at preventing or mitigating this debilitating disorder.

Of the approximately 50% of people with HIV (PWH) who develop peripheral neuropathy, 40% report experiencing DNP while the remaining 60% do not (1, 3). To date, it remains unclear why only some PWH develop this condition. Damage to peripheral nerves and clinical factors such as age, have all been implicated in development of HIV DNP. However, these factors alone appear insufficient to explain the manifestation and severity of this condition. Evidence that psychiatric and brain characteristics also play a role in HIV DNP (4–6) suggests that elucidation of central nervous system biomarkers could lead to a more complete mechanistic understanding of this condition. Recent advances in functional brain imaging have made it possible to investigate neurobiological contributions to HIV DNP. Although data specific to HIV DNP is scarce, functional neuroimaging of chronic pain, more broadly, implicates the role of altered functional connectivity (7, 8). Specifically, chronic pain studies have produced converging evidence of altered resting-state connectivity both with and within the default mode network (DMN), with overconnectivity between salience network (SN) and DMN regions (e.g., insula and MPFC) being identified as a central biomarker of chronic pain (9–17). However, it remains unclear the extent to which HIV DNP is associated with similar alterations in neural connectivity.

Data-driven approaches characterizing functional connectivity patterns show great promise in elucidating neurobiological mechanisms underlying clinical conditions. However, simple functional connectivity metrics, such as Pearson Correlation and distance measures, likely prohibit detection of the more complex and directional neural patterns that underlie neurologic conditions. Utilization of effective connectivity models in these populations could lead to improved understanding by providing directional and causal relationships between networks, rather than simple correlational associations (18, 19). A novel data-driven strategy called group iterative multiple model estimation (GIMME) appears particularly effective in deriving directed pathways and has been successful in separating clinically heterogenous populations (20, 21) and appears robust against problems associated with signal-to-noise ratio, hemodynamic response variability, and low sample sizes (21, 22). A modification, called subgrouping GIMME, can identify neural subtypes in small samples and outperforms other clustering methods (20). The current study utilized subgrouping GIMME with resting-state functional connectivity to identify DNP-associated functional connectivity patterns within SN and DMN regions that have been implicated in chronic pain conditions (i.e., medial prefrontal cortex [MPFC], posterior cingulate cortex [PCC], anterior cingulate cortex [ACC], insula, and thalamus) (10, 23). We hypothesized that HIV DNP would be associated with increased connectivity between SN and DMN regions (i.e., insula-MPFC) and decreased within DMN connectivity (i.e., MPFC-PCC).

## Materials and methods

### Participants

Participants were community-dwelling adult volunteers participating in research studies at the HIV Neurobehavioral Research Center (HNRC) at UC San Diego. Of note, the HNRC sample was approximately 80% male. 200 individuals were screened for eligibility in the study. 70 were deemed eligible and were included in the study, of whom 69 were male and one was female. Neuroimaging data was unusable for 7 (1 female, 6 male) of the 70 participants, primarily due to excessive motion artifact. Therefore, a total of 62 male participants between the ages of 39 and 70 (mean age = 58.2, SD = 7.8) with useable and complete data were included in the current analyses. See Table 1 for participant demographic and clinical characteristics. All participants were HIV seropositive and underwent an MRI scan, a structured clinical interview, and completed self-report measures to assess HIV, DNP, and psychiatric symptoms. Of the 62 participants, 30 endorsed and 32 denied trait DNP and 27 endorsed and 35 denied state DNP (24) on the day of MRI scanning. Potential participants were excluded based on the presence of a neurocognitive morbidity that is external to HIV illness, serious co-morbid medical condition unrelated to HIV, neurological confounds (e.g., head injury with loss of consciousness for greater than 30 min, seizure disorders, CNS neoplasms unrelated to HIV, MS), severe psychiatric disorder, current intoxication, or active abuse/dependence within last 30 days. In addition, participants were excluded if they had contraindications to MRI scanning such as pregnancy/breastfeeding, claustrophobia, or metal prosthesis or device. A power analysis was conducted using the PWR package in R and was based on recent findings from similar work by our group (25), which found large effect sizes (Cohen's *d* > 1.26). This analysis found that the minimum sample needed to detect the reported results with sufficient power (>0.80) was 11 per group. The UC San Diego institutional review board approved the use of humans for this study, all participants provided written informed consent prior to enrollment, and data were collected in accordance with all ethical standards as stipulated by the UC San Diego institutional review board-approved procedures.

**Table 1:** State distal neuropathic pain (DNP) subgroup demographic and clinical characteristics.

Variable	DNP* *+* * (*n* = 27) Mean [SD]	DNP− (*n* = 35) Mean [SD]	t/**χ**^**2**^	*df*	*p*
Age (years)	57.4 [7.5]	58.9 [8.1]	0.70	55.6	0.4855
Education (years)	14.3 [2.3]	15.1 [3.5]	1.11	55.1	0.27
Neuropathy severity (TNS)	12.3 [3.2]	6.79 [2.2]	7.53	42.8	<0.0001
Trait DNP severity (NEQ)	2.65 [1.2]	0.2 [0.5]	9.26	31.9	<0.0001
Paresthesia severity (NEQ)	2.0 [0.6]	1.2 [0.7]	3.65	57.0	0.0005
Non-neuropathic pain (BPI)	2.3 [2.3]	1.1 [2.0]	2.09	49.5	0.0416
Physical function (MOS)	59.9 [21.5]	69.8 [22.8]	1.72	55.4	0.0897
Depression (BDI-II)	14.9 [10.3]	8.1 [7.7]	2.79	44.4	0.0075
Anxiety (POMS)	10.3 [8.2]	5.65 [5.3]	2.52	40.1	0.0157
Fear of medical pain (FPQ)	22.34 [8.0]	22.76 [8.0]	0.20	54.1	0.8420
Pain Rumination (PCS)	7.38 [3.9]	6.03 [4.1]	1.29	55.0	0.1998

Note: TNS, Total neuropathy score; NEQ, neuromedical exam questionnaire; BPI, brief pain inventory; MOS, medical outcomes study; BDI, beck depression inventory; POMS, profile of mood states; FPQ, fear of pain questionnaire; PCS, pain catastrophizing scale; SD, standard deviation; *t*, *t*-statistic; *χ*^2^, chi square statistic; df, degrees of freedom; *p*, *p*-value.

### Clinical measures

Physicians and nurses trained in neurological AIDS disorders performed a standardized, targeted neurological examination to evaluate HIV neuropathy signs, including diminished ability to recognize vibration and reduced sharp-dull discrimination in the feet and toes or reduced ankle reflexes. The presence of at least two signs bilaterally was considered to be evidence of HIV neuropathy. Neuropathy symptoms also were assessed in the legs, feet, and toes and included bilateral neuropathic pain (burning, aching, or shooting), paresthesia, and loss of sensation. Using the Neuromedical Exam Questionnaire, clinicians classified trait neuropathic pain into the following 5 severity levels: none, slight (occasional, fleeting), mild (frequent), moderate (frequent, disabling), and severe (constant, daily, disabling, requiring analgesic medication or other treatment). At the time of the MRI brain scan, current state DNP was assessed using the Gracely Pain Scale (GPS). Those who reported state DNP (i.e., GPS >0, *n* = 27) were classified as DNP+, whereas those who did not state DNP (i.e., GPS = 0, *n* = 35) were classified as DNP−. For exploratory analysis, current state non-neuropathic pain (NNP) symptoms were also investigated using the Brief Pain Inventory (BPI). Those who reported state DNP (GPS >0) and/or NNP (BPI >0) were classified as any pain positive (AP+) whereas those who did not endorse DNP or NNP were classified as AP−. Psychiatric symptom data was collected *via* administration of the Beck Depression Inventory (BDI; depression) and the Profile of Mood States (POMS; anxiety and tension). Data with respect to fear of medical pain was collected *via* the Fear of Pain Questionnaire (26, 27) and data with respect to pain rumination was collected *via* the Pain Catastrophizing Scale (28).

### MRI acquisition and preprocessing

Imaging data were acquired on a GE Discovery MR750 3T whole body system with a body transmit coil and an 8-channel receive-only head coil at the University of California San Diego Center for Functional MRI. The structural brain sequence consisted of a high-resolution T1-weighted Fast Spoiled Gradient Recall (3D FSPGR) scan: 172 1.2 mm contiguous sagittal slices, FOV = 240 mm, TR = 8 ms, TE = 3.1 ms, flip angle = 8, TI = 600 ms, 256 × 192 matrix. The 8-minute functional scan was acquired using a T_2_*-weighted echo planar image (EPI) sequence (matrix = 64 × 64; 30 axial slices; in-plane resolution = 3.75 × 3.75 × 4; TR = 1.5s; TE = 30; flip-angle = 80°). Imaging preprocessing was conducted using a combination of AFNI (29) and ANTsR (https://github.com/ANTsX/ANTsR). T1 images were inhomogeneity-corrected to enhance registration using the N4 bias correction method (30). Each subject's T1 was brain extracted, diffeomorphically registered to the 1mm mni_icbm152_nlin template, and segmented using ANTS (31, 32). For preprocessing of the functional images, the first three EPI volumes were removed to allow for scanner equilibration. Next, EPI images were despiked, slice-time corrected, outliers were truncated and N4 biased corrected. Next, EPI images were temporally whitened to reduce bold-signal temporal autocorrelation, motion corrected, and noise components were removed using CompCor (33), spatially smoothed using Perona-Malik anisotropic diffusion (34), and band-pass filtered (0.001–0.01 Hz). Corrected EPI images were coregistered to the individual standardized T1 image using SyNBold. Forward and inverse registration transformations were concatenated and applied in a single step to minimize excess blurring of images. DVARs (>2.5 SD) and framewise displacement (FD >0.3 mm) were used to remove subjects due to excess motion.

### Resting-state networks

Mean BOLD time series were extracted from the average activity across voxels using five MNI regions of interest (ROIs; 6 mm radius) within the MPFC (*x* = −4, *y* = 58, *z* = 2), PCC (*x* = 2, *y* = −56, *z* = 26), ACC (*x* = 2, *y* = 36, *z* = 22), thalamus *x* = −10, *y* = −24, *z* = −2), and left insula (*x* = −38, *y* = 16, *z* = −12) based on prior research (10, 23). These five ROIs were then entered into a subgrouping GIMME, comparing those who reported DNP to those that did not (DNP+/−). Exploratory analyses (see Supplementary) also used subgrouping GIMME to compare those who reported any-pain (AP+) to those who did not (AP−).

### Statistical analyses

Effective connectivity analyses identifying the functional connectivity differences that characterize pain subgroups (DNP+/DNP−) were conducted using GIMME, a freely distributed package in R (package: *gimme*), which models the directed functional connectivity of fMRI BOLD signal from predefined brain ROIs and identifies patterns at the individual and group level (21, 35). Correlation matrices were created for each of the individual's ROI time series within the MPFC, PCC, ACC, left insula, and thalamus. GIMME first detects the signal and filters out noise across individuals, to create a group-level map of contemporaneous and lagged directed connections that are common for the majority of individuals, while allowing for the structure of connectivity maps to be person-specific. The probability of detecting an effect across all individuals was set at 75% (21, 35). Then, individual-level paths that will improve the model were freed, these paths were selected based on how many individuals' models would significantly improve. The paths represent how the relationship between two brain regions are influenced by the grouping variable. Then the common model was pruned by removing paths which are no longer acceptable; paths chosen earlier in the process were reevaluated because they had not yet been compared to all selected paths. Once these nonsignificant paths were removed, the GIMME model and a confirmatory model was fit. The final model for each individual included a partial connectivity map that was common across all individuals. Multiple comparisons are corrected for within GIMME using the lavaan package in R. Resulting connectivity strengths were reviewed for univariate outliers (>3SDs) and adjusted using winsorizing as needed prior to analyzing brain-behavior relationships.

*t*-tests and chi-square tests were used to compare GIMME subgroups on clinical variables (i.e., total peripheral neuropathy severity, DNP (state and trait), non-neuropathic pain, paresthesia, depression, anxiety, fear of medical pain, pain rumination) and demographic variables (i.e., age, education) and results were considered significant at *p* < 0.0045 (Bonferroni corrected). Post hoc analyses also explored associations of unique connectivity by group with clinical variables, statistically adjusting for demographic variables (i.e., age, education) and results were considered significant at *p* < 0.0055 (Bonferroni corrected).

## Results

Final model fit indices indicated excellent fit across individuals, CFI = 0.97 and SRMR = 0.03. GIMME identified common and unique network effective connectivity patterns across the two subgroups.

### Clinical characterization of subgroups

The DNP+ group demonstrated higher overall peripheral neuropathy severity (*t* = 7.53, *p *< 0.0001), trait DNP severity (*t* = 9.26, *p* < 0.0001), paresthesia severity (*t* = 3.65, *p* = 0.0005), and depression scores (*t* = 2.79, *p* = 0.0075), when compared to DNP− (see Table 1).

### Unique pathways

**Figure 1:**
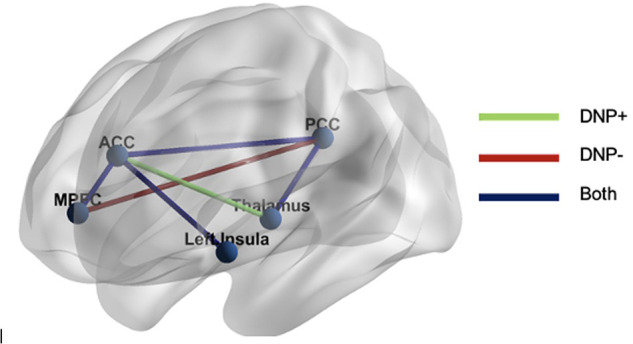
GIMME results. The DNP* *+* *group was characterized by functional connectivity between the thalamus and ACC, whereas the DNP− group was characterized by connectivity between the MPFC and PCC. Both groups demonstrated connectivity between the MPFC and ACC, ACC and PCC, ACC and insula, and PCC and thalamus. Note: MPFC, medial prefrontal cortex; ACC, anterior cingulate cortex; PCC, posterior cingulate cortex.

Examination of subgroup connectivity revealed that DNP+ group was characterized by a unique pathway between the thalamus and ACC (*β*** **= 0.348, *Z* = 5.778, *p* = 0.052), whereas the DNP− group was characterized by a unique pathway between the MPFC and PCC (*β*** **= 0.325, *Z* = 7.019, *p* = 0.024; see Table 2). Connectivity between ROIs within each subgroup are illustrated in Figure 1.

**Table 2:** Path estimates and Z-scores.

Path	*β* Mean	S.E.	Z	*p* value
**Common Paths**				
LINS to ACC	0.6788	0.0428	16.9217	0.0018
MPFC to ACC	0.6347	0.0415	16.4678	<0.0001
PCC to ACC	0.6715	0.0417	17.0863	<0.0001
Thalamus to PCC	0.4272	0.0586	8.1640	0.0262
**Unique Paths**				
*DNP− group*				
MPFC to PCC	0.3258	0.0479	7.0199	0.0243
*DNP + group*				
Thalamus to ACC	0.3482	0.0623	5.7786	0.0522

Note: *β* Means, S.E.s, Z, and *p* values are computed as an average across subjects; MPFC, medial prefrontal cortex, ACC, anterior cingulate cortex, LINS, left insula, PCC, posterior cingulate cortex.

### Common pathways

**Figure 2:**
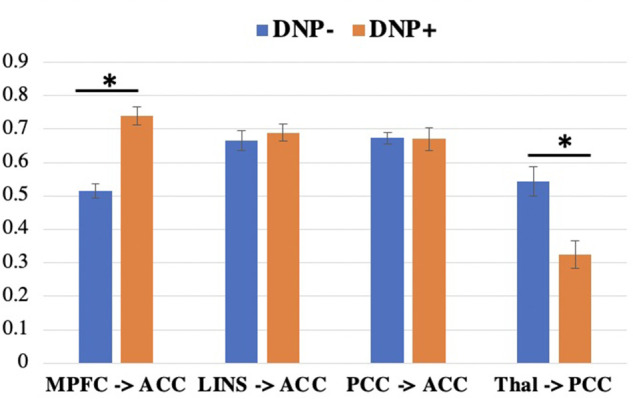
Connectivity subgroup differences in group-level paths. DNP+ had a greater MPFC to ACC path and a lower thalamus to PCC path, compared to subgroup DNP−. *p <* *0.05 (uncorrected). Note: MPFC, medial prefrontal cortex; ACC, anterior cingulate cortex; LINS, left insula; PCC, posterior cingulate cortex; Thal, thalamus.

Across all subjects, a series of four contemporaneous directed connectivity pathways were identified. These connectivity paths consisted of directed paths from the MPFC to the ACC, from the LINS to the ACC, from the PCC to the ACC, and from the thalamus to the PCC. Although these paths are common across the two groups, group differences in strength in these networks emerged in two out of the seven networks. Specifically, DNP + had significantly greater MPFC to ACC connectivity (*t* = 4.6093, *p* < 0.001; Figure 2) and lower thalamus to PCC connectivity compared to DNP− (*t* = 2.52, *p* = 0.0144; Figure 2).

### Exploratory analysis

Post hoc analyses showed that connectivity between the MPFC and PCC and between the thalamus and ACC was not associated with clinical variables, including neuropathy severity, trait DNP severity, paresthesia severity, non-neuropathic pain, physical function, depression, anxiety, fear of medical pain, and pain rumination (all *p*s >0.005). Please see Supplementary showing similar unique pathway results when comparing groups on AP + (DNP and/or non-neuropathic pain) vs. AP−.

## Discussion

The present study is the first to report findings of DMN and SN resting-state functional connectivity patterns associated with DNP among those with HIV. Although we did not observe increased connectivity between SN and DMN regions, our additional hypothesis of decreased within DMN connectivity was indeed supported by the data. Specifically, those who endorsed DNP showed less connectivity between the MPFC and PCC and greater connectivity between the ACC and thalamus, relative to those who denied DNP. Taken together, these results implicate weaker within-DMN connectivity and stronger within-SN connectivity as potential mechanisms underlying the development and/or maintenance of HIV DNP. We speculate that these findings could reflect differential brain responses to pain, with important implications for the development of novel treatments aimed at preventing or mitigating HIV DNP.

Relatively less within-DMN (i.e., MPFC and PCC, Figure 1) connectivity in the DNP+ group compared to the DNP− group is largely consistent with emerging evidence within the broader chronic pain literature suggesting that altered within-DMN resting-state connectivity plays a critical role in the experience of chronic pain. However, it should be noted that the literature is somewhat mixed, with evidence of both higher (10, 11, 14, 17) and lower (9, 10, 17, 36) DMN connectivity in chronic pain, perhaps reflecting different aspects of the DMN regulatory response to pain, as well as chronic pain heterogeneity. In this context, the current findings lend further support to the notion of pain associated decreases in within-DMN connectivity. More specifically, we speculate that relatively less MPFC-PCC connectivity in those experiencing DNP may reflect a reduced ability to engage in mind wandering (i.e., attention fluctuating away from the present sensory environment), as has been suggested in other studies of chronic pain (37). More specifically, in the setting of a painful experience in which ascending nociception mechanisms are engaged, the salience network is thought to help the brain disengage from attention networks focusing on pain and to engage the DMN to focus on something other than pain (i.e., mind wandering). In this process, the brain is also thought to activate endogenous analgesic activity within the descending pain modulatory system (e.g., MPFC, ACC, periaqueductal gray [PAG]) as a way to decrease pain signal during mind wandering. In contrast, decreased DMN activity in the setting of a painful experience is thought to enhance the salience of pain (38). Therefore, it is possible that our analysis revealed a subgroup experiencing state DNP that is primarily manifested by decreased DMN connectivity at rest, reflecting an impaired ability to mind wander away from the experience of pain, and thus increase the salience of pain. In fact, the latter notion of increased salience of pain appears to be supported by our additional finding of greater within SN connectivity (i.e., ACC and thalamus) in the DNP+ group. The SN processes “bottom-up” aversive or other salient attention-demanding stimuli or experiences, such as pain, and shows chronic pain related alterations (39). The ACC and thalamus are thought to be core regions of pain processing modulation, involved in the motivational and affective aspects of pain (40, 41), with ACC being interoceptive motor control region (42). More specifically, evidence suggests that in response to pain, the ACC engages lower parts of the descending pain control system (i.e., PAG, hypothalamus, rostral ventromedial medulla), which in turn exert an opioid-dependent inhibitory influence on spinal nociceptive processing, reducing nociceptive input to thalamic and cortical regions, and ultimately leading to a reduced pain experience (43). As such, it is possible that our observation of greater relative connectivity between the ACC and thalamus in DNP+ could reflect increased activation of the descending analgesic pathway in the setting of pain. However, it should be noted that this pathway describes pain facilitation *and* pain inhibition, and we are unable to differentiate between the two using the current data. Perhaps more fitting with the current data, we point to results from animal research that support the notion of a specific input-output pathway involving medial dorsal thalamic inputs to subcortically-projecting neurons within the ACC that contributes to pain-related aversion (44). Therefore, our finding of a DNP-related increase in connectivity between the ACC and thalamus could reflect altered pain-related aversion and/or pain facilitation and/or ineffective inhibition, though future investigations are required to determine which interpretation is most accurate.

Taken together, the current findings implicate reduced within DMN connectivity (i.e., MPFC and PCC) and increased SN connectivity (i.e., ACC and thalamus) as potential manifestations of adaptation to pain from neuropathy and/or mechanisms underlying the development/maintenance of HIV DNP. Although we speculate how the current findings potentially reflect differential responses to pain (e.g., mind wandering, pain aversion, pain facilitation/inhibition), future research is needed to test these hypotheses directly. Of note, the similarities of findings from additional analyses presented within the supplement regarding subgroups defined by the presence of any pain (AP), defined as both neuropathic and non-neuropathic (see Supplementary) suggest that these DNP-related functional connectivity patterns may extend to the experience of pain more broadly. In this broader context, it should be noted that several studies have reported partial reversals of DMN connectivity changes with reductions in clinical pain after treatment (16, 36) or augmentation of DMN changes with exacerbation of clinical pain (13, 15, 45). Therefore, the current results could have important implications for DNP treatment, as intervention strategies that have been shown to improve connectivity within SN and DMN regions (e.g., mindfulness-based meditation, physical activity interventions, cognitive interventions) could be utilized among those diagnosed with HIV to prevent the development of, or lead to improvement in DNP symptoms.

Limitations of the current study include the use of a cross-sectional design, which restricted our ability to draw causal conclusions. Furthermore, our study was restricted to those with HIV, thus precluding the evaluation of the uniqueness of brain connectivity in this specific disease. In addition, we examined an all-male sample which prevents generalizing our findings to the female population, which would be important to address in future work. It is also important to note that our sample had relatively high levels of education, and although this demographic factor did not differ between groups, its limited range may reduce the generalizability of these findings. Moreover, our overall sample size was relatively small (*n* = 63) but is comparable with other recent studies using rs-fMRI. Furthermore, GIMME has been shown to be robust with small sample sizes, which suggests that this is an ideal method for investigations with challenging-to-recruit patient populations. Nonetheless, future work with larger samples will be required to determine replicability of the subtypes observed in our study and to probe the unique within-subgroup connectivity profiles. With regard to strengths, it should be noted that this is the first study to examine resting state functional connectivity patterns in HIV DNP. Additional strengths include our use of GIMME, which offers improvement over simple functional connectivity models (e.g., Pearson Correlation, distance measures) by providing directional and causal relationships between networks (18, 19, 46). Our study also benefitted from the use of multiple clinical measures to assess peripheral neuropathy symptoms and signs, as well as DNP and non-neuropathic pain severity. Assessment of DNP and non-neuropathic pain directly before obtaining neuroimaging data also represents a strength, as there may be different brain mechanisms underlying the state and trait aspects of pain (24).

## Conclusions

We report novel evidence of unique functional connectivity patterns associated with HIV DNP. Findings implicate reduced within DMN connectivity (MPFC and PCC) and increased within SN connectivity (thalamus and ACC) as potential manifestations of adaptation to pain from neuropathy and/or mechanisms underlying the development/maintenance of HIV DNP, perhaps reflecting different brain responses to pain (e.g., decreased mind wandering, increased pain aversion, pain facilitation/inhibition), though future research is needed to test these hypotheses more directly.

## Data Availability

The raw data supporting the conclusions of this article will be made available by the authors, without undue reservation.
