# Supplementary material for: Distal neuropathic pain in HIV is associated with functional connectivity patterns in default mode and salience networks
Source: Front Pain Res (Lausanne). 2022 Oct 12;3:1004060. doi: 10.3389/fpain.2022.1004060 (PMC9596968; doi:10.3389/fpain.2022.1004060)
Supplement: Supplementary file 1 [file DataSheet1.docx]

**Supplementary Materials**

Results from exploratory subgrouping GIMME showed that the any-pain (AP+) group was characterized by functional connectivity between the thalamus and ACC, whereas the no-pain (AP-) group was characterized by connectivity between the MPFC, PCC, and thalamus. Both groups demonstrated connectivity between the MPFC and ACC, PCC and ACC, and insula and ACC (see Figure S1 and Table S1). The AP+ group demonstrated higher peripheral neuropathy severity and trait DNP severity, when compared to AP- (see Table S2).


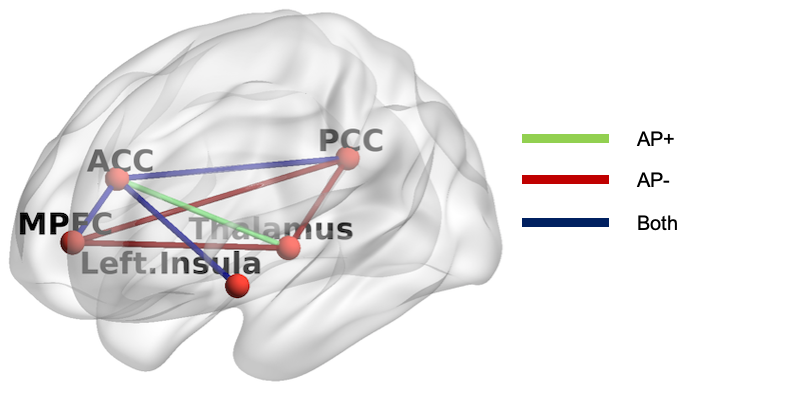


**Figure S1.** The AP+ group was characterized by functional connectivity between the thalamus and ACC, whereas the AP- group was characterized by connectivity between the MPFC, PCC, and thalamus. Both groups demonstrated connectivity between the MPFC and ACC, ACC and PCC, and ACC and insula.

| **Table 2. Path Estimates and Z-scores** | | | | |  |
| --- | --- | --- | --- | --- | --- |
| **Path** | **β Mean** | **S.E.** | **Z** | ***p value*** | |
| **Common Paths** | | | | | |
| LINS to ACC | 0.6873 | 0.0428 | 17.1562 | 0.0019 | |
| MPFC to ACC | 0.6383 | 0.0408 | 16.7522 | 0.0011 | |
| PCC to ACC | 0.6559 | 0.0416 | 16.7429 | 0.0009 | |
| **Unique Paths** | | | | | |
| *AP- group* | | | | | |
| MPFC to PCC | 0.3466 | 0.0470 | 7.4917 | 0.0237 | |
| Thalamus to MPFC | 0.1958 | 0.0699 | 3.2514 | 0.0732 | |
| Thalamus to PCC | 0.4126 | 0.0716 | 5.9099 | 0.0552 | |
| *AP+ group* | | | | | |
| Thalamus to ACC | 0.5420 | 0.0463 | 12.9852 | 0.0212 | |

| **Table S2. Any-pain (AP) subgroup demographic and clinical characteristics** | | | | | |  |  |
| --- | --- | --- | --- | --- | --- | --- | --- |
| **Variable** | **AP+ (n=40)**  **Mean [SD]** | **AP- (n=22)**  **Mean [SD]** | **t /**$\boldsymbol{\chi}\boldsymbol{2}$ | ***df*** | ***p*** | |  |
| Age (years) | 57.2 [7.9] | 59.9 [7.4] | 1.32 | 45.7 | 0.1904 | |  |
| Education (years) | 14.8 [2.9] | 14.9 [3.6] | 0.09 | 34.1 | 0.9281 | |  |
| Neuropathy severity (TNS) | 10.7 [4.1] | 6.6 [1.8] | 5.47 | 57.9 | <0.0001** | |  |
| Train DNP severity (NEQ) | 1.9 [1.5] | 0.1 [0.4] | 7.10 | 48.7 | <0.0001** | |  |
| Paresthesia severity (NEQ) | 1.8 [0.7] | 1.2 [0.7] | 2.56 | 44.5 | 0.01391 | |  |
| Physical function (MOS) | 62.5 [21.1] | 70.5 [23.9] | 1.30 | 38.9 | 0.1998 | |  |
| Depression (BDI-II) | 13.3 [9.7] | 8.1 [8.6] | 2.15 | 48.0 | 0.0360 | |  |
| Anxiety (POMS) | 8.7 [7.4] | 5.9 [5.8] | 1.62 | 52.4 | 0.1095 | |  |
| Fear of medical pain (FPQ) | 22.6 [8.4] | 22.3 [7.0] | 0.15 | 53.2 | 0.8802 | |  |
| Pain Rumination (PCS) | 7.1 [3.9] | 5.7 [4.0] | 1.30 | 45.6 | 0.1983 | |  |
| Note: TNS= Total Neuropathy Score; NEQ= Neuromedical Exam Questionnaire; BPI= Brief Pain Inventory ; MOS= Medical Outcomes Study; BDI= Beck Depression Inventory; POMS= Profile of Mood States; FPQ= Fear of Pain Questionnaire; PCS=Pain Catastrophizing Scale; SD= standard deviation; t= t-statistic; $\chi2$= chi square statistic; df= degrees of freedom; p= p-value; *significant at p<0.0045, **significant at p<0.00045 | | | | | | | |
